# Peripheral and cerebrospinal fluid inflammatory markers in sporadic amyotrophic lateral sclerosis an exploratory cross-sectional association study

**DOI:** 10.1007/s10072-026-09480-9

**Published:** 2026-10-17

**Authors:** Agnieszka Wełnicka-Wesołowska, Karolina Łuczkowska, Przemysław Nowacki, Edyta Paczkowska, Agnieszka Meller, Karolina Machowska-Sempruch, Bogusław Machaliński, Monika Gołąb-Janowska

**Affiliations:** 1 Department of Neurology, Pomeranian Medical University, Unii Lubelskiej 1 Str 71-252 Szczecin, Szczecin, Poland; 2 Department of General Pathology, Pomeranian Medical University, Szczecin, Poland

**Keywords:** Amyotrophic lateral sclerosis - pathogenesis, Inflammation of the central nervous system, Neuroinflammation, TNF receptor 1, Biomarker

## Abstract

Amyotrophic lateral sclerosis (ALS) is a progressive neurodegenerative disorder with an ambiguous etiology, whose onset may be linked to innate immune system activation outside the central nervous system (CNS). We investigated the association between peripheral blood and CNS inflammatory parameters in 45 sporadic ALS patients compared to 30 controls, analyzing peripheral blood tests (C-reactive protein [CRP], blood count, fibrinogen) and cerebrospinal fluid (CSF) immunological measures (Tumor Necrosis Factor alpha [TNF-α], TNF receptor type 1 [TNFR-1], CRP) alongside clinical characteristics. Results revealed significantly higher concentrations of TNFR-1 in the CSF of ALS patients compared to the control group (1,029.5 ± 176.0 vs. 846.1 ± 167.5 pg/ml; *p* < 0.0001). This difference remained significant in multivariable logistic regression adjusting for age and sex (OR 1.82 per 100 pg/ml increment, 95% CI 1.26–2.63; *p* = 0.0015). In an exploratory ROC analysis, CSF TNFR-1 discriminated patients from controls with an AUC of 0.78 (95% CI 0.67–0.88). TNFR-1 correlated with age (*R* = 0.32, *p* = 0.038), associations with disease severity and a lower concentration in bulbar-onset disease were exploratory and not robustly significant. Plasma and CSF CRP concentrations did not differ significantly between groups, but strongly correlated with each other in both ALS (*R* = 0.83) and control (*R* = 0.82) cohorts, indicating a disease-non-specific relationship. CSF TNFR-1 is independently associated with sporadic ALS and warrants further investigation as a candidate disease-associated inflammatory biomarker. Given the limitations, our findings do not prove diagnostic accuracy or causality. Longitudinal studies with disease controls and blood-CSF barrier integrity measures are needed.

## Introduction

Amyotrophic lateral sclerosis (ALS) remains a condition of ambiguous etiology and pathogenesis [1, 2]. The disorder has a progressive course, leading to disability and death, usually within 2–5 years of diagnosis, due to respiratory failure or complications of immobilization [2]. Available therapies remains unsatisfactory and survival time is short [3].

Growing evidence suggests that the onset of the pathological process may occur outside the central nervous system (CNS) and is linked to systemic inflammation and innate immune activation [1, 4]. In ALS, neurodegeneration is consistently accompanied by CNS inflammation - microglial activation and infiltration of T lymphocytes and macrophages. Studies in *SOD1 gene* in mice show that the markers of inflammation, including microglial and complement activation, precede the onset of degeneration [4].

Activated microglia secrete proinflammatory cytokines and reactive oxygen species/nitrogen species [5] and increase blood-brain barrier (BBB) permeability, allowing infiltration of peripheral inflammatory cells, that may contribute to neuronal damage [6]. This is associated with a systemic immune response [7]. Under normal conditions this reaction is quenched by anti-inflammatory, tissue remodelling signals; in neurodegenerative diseases, this control is lost [8], and chronically activated glia, which initially act protectively, eventually cause neuronal damage [9]. Their current function depends on interactions with surrounding immune cells [6, 10]. Age further predisposes microglia to a pro-inflammatory state, lowering the threshold at which external stimuli induce chronic neuroinflammation [11, 12].

Several ALS-associated genes converge on inflammatory pathways. Microglia expressing SOD1(G93A) are more neurotoxic than wild-type microglia [6, 13] and reducing *SOD1* expression in these cells prolongs survival in transgenic mice [14]. The C9orf72 hexanucleotide repeat expansion, the most common mutation in ALS, is associated with an increased inflammatory response and impaired clearance of aggregated proteins, accelerating neurodegeneration [15]. Mutant TAR DNA-binding protein 43 (TDP-43) promotes expression of proinflammatory factors, including Tumor Necrosis Factor alpha (TNF-α) or interleukin 1β [16]. Both the C9orf72 expansion and TDP-43 mutations are additionally associated with impaired interferon regulation, which may drive systemic inflammation. Other ALS-associated genes, including *OPTN*,* SQSTM1*,* TBK1* and *VCP*, are also implicated in inflammatory pathways [15]. Modulating macrophage/microglia activity has shown partial success in slowing disease progression in selected patients [17]; peripheral macrophages modification can affect central microglial activity and prolong survival [18].

The primary objective of this study was to evaluate the association between peripheral blood inflammatory biomarkers, which indirectly reflect overall immune activity [2, 19], and cerebrospinal fluid (CSF) inflammatory parameters in ALS patients compared with controls. Literature addressing this specific peripheral-CSF relationships remains sparse. Although elevated CSF proinflammatory cytokines in ALS are well documented [2, 20–22] and peripheral markers have prognostic value for disease progression [23, 24], simultaneous analysis of both compartments is lacking, and to our knowledge no prior study has examined the direct relationship between C-reactive protein (CRP) kinetics and CSF parameters in ALS. TNF-α and its respective receptors, including TNF receptor 1 (TNFR-1) have previously been studied in the systemic circulation, where serum concentrations correlate with disease duration [25–27]. This study’s pre-specified primary analysis was the comparison of CSF TNFR-1, TNF-α and CRP between ALS patients and controls and their relationship to peripheral inflammatory parameters; associations with clinical phenotype, severity, and disease progression rate were treated as exploratory, hypothesis-generating secondary analyses.

## Materials and methods

### Study population

The study group consisted of 45 patients with a diagnosis of sporadic ALS, based on the modified El Escorial criteria [28]. Participants were consecutively recruited between 2018 and 2019 from a cohort enrolled in a clinical trial (STRATEGMED1/234261/2NCBR/2014), that specified an upper limit of 65 years as an eligibility criterion, with no excluded participants from parent trial. This age restriction, while methodologically necessary given the parent trial’s criteria, limits generalizability to the wider ALS population, whose peak incidence occurs between 50 and 70 years.

Each patient was interviewed using an original form recording age, sex, age at onset, disease duration and phenotype, and then underwent a neurological and internal medical examination, and assessment with the modified Norris scale (assessing the severity of the disease), the Amyotrophic Lateral Sclerosis Functional Rating Scale-Revised (ALSFRS-R) and the ALSAQ-40 quality-of-life scale. Exclusion criteria were:

- age under 18 or over 65,
- inability to give informed consent to participate or features of dementia,
- comorbidities that could affect the parameters assessed in this study,
- medications affecting bone marrow function (related to parent trial).

The control group consisted of 30 individuals evaluated in the Neurology Emergency Room for headache, with no evidence of chronic neurological disease. Each underwent an interview and neurological and internal medical examination. Patients with clinical or laboratory evidence of an active infectious, autoimmune or systemic inflammatory condition at presentation were excluded, in addition to:

- age under 18,
- abnormal general CSF examination.

For both groups, peripheral blood tests (full blood count, CRP, fibrinogen) and immunological, fluorometric CSF tests (TNF-α, TNFR-1 and CRP) were performed using the Luminex platform (Luminex Corporation, USA). The CSF was centrifuged in an Eppendorf centrifuge at 2,000 rpm for 10 min; the resulting supernatant was then stored at -80 °C until analysis. Samples were thawed only once immediately prior to testing. Assays for both the study and control groups were conducted in a single run, and the laboratory personnel were not blinded to the group assignments.

### Statistical analysis

Continuous variables were tested for normality in each group with the Shapiro-Wilk test. Normally distributed variables (e.g., TNFR-1) are reported as mean ± SD and compared with Student’s t-test, ANCOVA, or multivariable regression as appropriate. Non-normally distributed variables (CRP in plasma and CSF, fibrinogen) are reported as median [interquartile range, IQR] and compared with the Mann-Whitney U test. Categorical variables are reported as percentages and compared with the χ² test.

Associations between continuous variables were assessed firstly by dichotomizing at the median and secondly with Spearman’s rank correlation. The relationship between CSF TNFR-1, age, sex, and group (ALS vs. control) was modelled with multivariable logistic regression, reporting odds ratios (OR) with 95% confidence intervals. The ability of CSF TNFR-1 to discriminate ALS from controls was assessed with receiver operating characteristic (ROC) analysis, reporting the area under the curve (AUC) with a bootstrap 95% CI (2,000 resamples); the associated Youden-optimal cut-off is reported as an exploratory, non-validated estimate derived and evaluated in the same sample, not as a validated diagnostic threshold. An ALSFRS-R–based progression rate was calculated for each ALS patient as (48 − ALSFRS-R) / disease duration in months. The primary analysis was the comparison of CSF TNFR-1, TNF-α and CRP between ALS and control groups and their correlation with peripheral inflammatory markers. Associations with clinical phenotype, disease severity, and progression rate were pre-specified as exploratory. Because several exploratory comparisons were performed, a Benjamini-Hochberg false discovery rate (FDR) correction was applied to the exploratory correlations reported; this multiplicity adjustment was not part of the original analysis plan and is noted as a limitation. All statistical analyses were performed in STATISTICA 13.3 and Python.

The study was positively evaluated by the Bioethics Committee of the Pomeranian Medical University in Szczecin (resolution no. KB-0012/06/10/A-1 adopted on January 25, 2010, with subsequent consent for changes on January 27, 2020) and was conducted in accordance with the Helsinki Declaration. All participants signed informed consent.

## Results

### Descriptive characteristics

The study group included 53.4% men; aged 27–65 years (mean 53.8 ± 9.5). The control group included 56.6% women, aged 23–78 years (mean 47.3 ± 12.8). Groups did not differ in sex distribution (χ2 test, *p* = 0.540), but a statistically significant age difference was found (t-test, *p* = 0.014).

In the study group, disease duration ranged from 5 months to 10 years (mean 29.6 ± 26 months). Patients with the limb-onset predominated (77.8%, *n* = 35); bulbar-onset occurred in 22.2% (*n* = 10), no respiratory-onset phenotype was observed. On average, patients showed moderate deterioration in daily functioning (ALSFRS-R 29.2 ± 5.0) and more-than-moderate disease severity (Norris scale 85.6 ± 15.6 ). The quality of life was higher than moderate (ALSAQ-40 36.3 ± 16.1).

### Laboratory characteristics

Results of peripheral blood and CSF tests are presented in Tables 1 and 2. Mean total leukocyte count was within the normal range in both groups, but higher in controls than in ALS patients (*p* = 0.045). Neutrophil and lymphocyte counts and the neutrophil-to-lymphocyte ratio (NLR) did not differ statistically between groups. Plasma CRP did not differ significantly between groups (both markedly non-normally distributed; Mann-Whitney *p* = 0.68). A significantly higher fibrinogen levels were found among ALS patients (*p* = 0.011). CSF CRP, markedly right-skewed in both groups (Shapiro-Wilk *p* < 0.0001), was not significantly different between ALS (median 9,975 pg/ml, IQR 4,196–25,475) and controls (median 4,904 pg/ml, IQR 2,983–16,064; Mann-Whitney *p* = 0.089). TNF-α concentrations in both groups were below the assay’s limit of quantification (< 2.77 pg/ml) and could not be analyzed further. CSF TNFR-1, which did not deviate from normality in either group (Shapiro-Wilk *p* = 0.45 and *p* = 0.66), was significantly higher in ALS patients (1,029.5 ± 176.0 pg/ml) than in the control group (846.1 ± 167.5 pg/ml; unadjusted t-test *p* < 0.0001). This difference remained significant after adjustment for age and sex (multivariable logistic regression, OR 1.82 per 100 pg/ml increment, 95% CI 1.26–2.63, *p* = 0.0015).

**Table 1:** Comparison of peripheral blood test results between the study and control groups. Values are mean ± SD for normally distributed variables and median [IQR] for non-normally distributed variables (plasma CRP, fibrinogen); group comparisons use Student’s t-test or the Mann-Whitney U test accordingly

Parameter (unit)	Study group	Control group	Statistical significance (*p*)
leukocytes (G/L), mean ± SD	**6.81 ± 1.8**	**7.59 ± 1.83**	0.045
neutrophils (G/L), mean ± SD	**4.26 ± 1.4**	**4.79 ± 1.37**	0.127
lymphocytes (G/L), mean ± SD	**1.88 ± 0.63**	**2.04 ± 0.7**	0.343
NLR, mean ± SD	**2.5 ± 1.25**	**2.59 ± 1.14**	0.547
Plasma CRP (mg/l), median [IQR]	**0.74 [0.10–1.87]**	**0.92 [0.10–1.66]**	0.68 (MW)
fibrinogen (mg/dl), median [IQR]	**343.2 [288.5-398.6]**	**292.3 [254.4-324.5]**	0.012 (MW)

**Table 2:** Comparison of CSF test results between the study and control groups. TNFR-1 is reported as mean ± SD (normally distributed); CRP is reported as median [IQR] (non-normally distributed). TNF-α was below the limit of quantification (< 2.77 pg/mL) in all participants.

Parameter (unit)	Study group	Control group	Statistical significance (*p*)
CSF CRP (pg/ml), median [IQR]	9,975 [4,196 − 25,247]	4,904 [2,983 − 16,064]	0.089 (MW)
TNF-α (pg/ml)	< 2.77 (all)	< 2.77 (all)	-
TNFR-1 (pg/ml), mean ± SD	**1**,**029.52 ± 175.97**	**846.1 ± 167.51**	< 0.0001

### Relationship between assessed parameters

In a multivariable logistic regression with ALS-vs-control group as the outcome and TNFR-1, age, and sex as covariates (*n* = 73), CSF TNFR-1 was independently associated with ALS diagnosis (OR 1.82 per 100 pg/ml increment, 95% CI 1.26–2.63, *p* = 0.0015); neither age (OR 1.01 per year, 95% CI 0.95–1.06, *p* = 0.82) nor sex (OR 0.99, 95% CI 0.33–2.94, *p* = 0.99) was independently associated with group once TNFR-1 was included. In an exploratory ROC analysis, CSF TNFR-1 discriminated ALS from controls with an AUC of 0.78 (95% CI 0.67–0.88, bootstrap). The Youden-optimal cut-off (≈ 1,005 pg/ml) yielded 68% sensitivity and 83% specificity; because this threshold was derived and evaluated in the same, single-cohort sample, without an independent validation set or a disease-control comparison group, it should be regarded as hypothesis-generating and not as a validated diagnostic criterion.

Among ALS patients, higher CSF CRP concentrations were found in subgroups with higher neutrophil counts, lymphocyte counts, fibrinogen and plasma CRP. Plasma and CSF CRP were strongly correlated in both the ALS group (Spearman *R* = 0.83, *p* < 0.0001) and the control group (*R* = 0.82, *p* < 0.0001). Because this correlation was similar in both groups, it is interpreted descriptively as a robust, disease-non-specific relationship between systemic and CSF CRP. Additionally, a statistically significant correlation was found between NLR and plasma CRP, but only using a parametric method on median-split subgroups (*R* = 0.42, *p* = 0.004), which could not be confirmed using Spearman correlation on the continuous variables (*R* = 0.11, *p* = 0.47).

Regarding study group descriptors, we found significantly higher concentrations of CSF TNFR-1 in older ALS patients both in the median-split analysis (1,076.22 pg/ml ± 176.29 vs. 978.38 pg/ml ± 164.72, *p* = 0.035) and as a continuous variable (Spearman *R* = 0.32, *p* = 0.038). The association between higher TNFR-1 and greater disease severity (median-split Norris subgroups, *p* = 0.046) did not replicate when severity was analyzed as a continuous variable (*R* = − 0.12, *p* = 0.43). In the analysis of covariance, neither the severity of the disease (*p* = 0.482) nor the age (*p* = 0.054) of the patients had a statistically significant effect on TNFR-1 levels. An ALSFRS-R–based progression rate (mean 1.05 ± 0.81 points/month; median 0.77, IQR 0.55–1.23) was not significantly correlated with CSF TNFR-1 (*R* = 0.21, *p* = 0.18) or CSF CRP (*R* = 0.06, *p* = 0.68).

We additionally performed multivariable regression analysis, including phenotype alongside other covariates, where lower CSF TNFR-1 was associated with bulbar-onset disease (*p* = 0.023). However, the univariate comparison between bulbar-onset (*n* = 10; mean 971.1 ± 158.9 pg/ml) and limb-onset (*n* = 34; mean 1,046.7 ± 179.2 pg/ml) patients did not reach significance (t-test *p* = 0.237; Mann-Whitney *p* = 0.127; Cohen’s d = 0.43; mean difference 75.6 pg/ml, 95% CI − 47.9 to 199.0). Given the small bulbar subgroup (*n* = 10) and the number of comparisons performed, this association is reported as an exploratory, hypothesis-generating observation and should not be interpreted as evidence of phenotype-specific biology.

A Benjamini-Hochberg FDR correction applied to the main exploratory comparisons above (plasma–CSF CRP correlation in each group, age–TNFR-1 correlation, and the adjusted bulbar-onset association) showed that all associations that survived the continuous analysis remained significant after correction (adjusted *p* ≤ 0.043).

## Discussion

### Leukocytes

Total leukocyte counts were higher in controls than in ALS patients, whereas neutrophil and lymphocyte levels were comparable; these results may be affected by the nature of the control group, which cannot be considered entirely healthy. Previous studies on leukocytes in ALS are inconsistent: some report elevated counts of all leukocyte types [29], others do not [30, 31].

Elevated levels of neutrophils in patients with ALS were reported and, less frequently, higher NLR, which correlated with faster disease progression, worse respiratory function and hypermetabolism [29, 32–34], and with shorter survival, especially in women [33, 35]. Pre-diagnostic elevations in neutrophils/NLR have been linked to higher subsequent ALS risk [36]. One study, based on the Mendelian randomization method, found the opposite direction of effects - genetically determined increased number of neutrophils was associated with a reduced risk of ALS [30].

Lymphocytes findings are also mixed, showing their increased number [37–39] or no difference compared to healthy individuals [40]. Most studies report a decreasing trend associated with faster progression and cognitive impairment [38, 41]. Regarding specific lymphocyte subsets (such as CD4 + and CD8 + T cells), existing literature has also yielded conflicting results.

In our cohort, NLR did not differ between groups, in line with Keizman et al. [23] regarding low-grade systemic inflammation. In the work of Leone et al. [42], higher NLR values were correlated with a faster rate of disease progression and higher mortality – the cut-off point here was 2.315, after which the mortality rate was twice as high. Wei et al. [43] and Choi et al. [44], similarly, confirmed the association of higher NLR with respiratory failure, faster disease progression and shorter survival, although they also noted the older age of disease onset in these groups.

### CRP

We did not find a significant difference in plasma or CSF CRP between ALS patients and controls. Prior studies were mixed, but with most reporting higher CRP in ALS [19, 23, 45, 46], also in CSF [47], including specifically in individuals with the C9orf72 hexanucleotide repeat expansion [48]. Higher pre-diagnostic CRP has been associated with increased ALS risk [49]. A 2022 systematic review [50] of over 5 thousand individuals found higher plasma CRP concentration in ALS overall.

In our ALS patients, higher CSF CRP was associated with higher peripheral inflammatory parameters, and plasma and CSF CRP were strongly correlated (Spearman *R* = 0.83, *p* < 0.0001). This correlation, however, was equally strong in controls, and we therefore interpret it descriptively as a disease-non-specific relationship between systemic and CSF CRP rather than as evidence of ALS-specific blood–CSF barrier disruption or central-peripheral inflammatory “crosstalk”. Prior studies suggested the existence of a relationship between peripheral inflammation and local inflammation in the CNS in neurodegenerative diseases [51], and CRP being a a potential ALS marker [52]. Systemic inflammation can disrupt the BBB, causing white blood cell infiltration into the CSF [53]. Additionally, local production of TNF-α and reactive oxygen species by glia and neurons may damage this barrier, promoting CRP penetration into the CNS [18]. Previously, local CNS production of CRP was linked only to pathological conditions [54], as low peripheral CRP concentrations were believed not to cross the BBB. However, plasma CRP levels above a 2.5 mg/l threshold can increase BBB permeability [55], which applied to 20% of our control group.

In the available literature, we found no studies assessing the relationship between CRP and CSF parameters in ALS, but plasma-CSF CRP correlations in non-ALS populations have been reported previously [56, 57], consistent with our interpretation that this relationship is not ALS-specific.

Studies on the relationship between CRP and the phenotype and symptoms of ALS are scarce. Contrary to some prior reports [19, 23, 45, 50, 58, 59], we did not find a correlation between plasma CRP and functional status in our patients.

### Fibrinogen

Higher plasma fibrinogen in ALS patients than controls, as found here, is consistent with prior reports [23] and with neuropathological evidence of perivascular and gray-matter fibrin deposition in ALS [60, 61]. Fibrinogen influences microglial activation, oxidative stress, mitochondrial dysfunction and neuroinflammation, mechanisms implicated in ALS pathogenesis. A 2021 study of γ-fibrinogen isoform (physiologically accounting for 8% of the total amount of this protein) found an independent association between higher levels and longer survival, suggesting a possible protective, anti-inflammatory microglial shift [62]; whether this applies to total fibrinogen, as measured here, remains to be tested.

### Tumor necrosis factor and its receptor

TNF-α concentrations were below the assay’s limit of quantification in both groups and no biological conclusions regarding TNF-α can be drawn from the present data. Prior studies show elevated TNF-α levels associated with ALS duration, in autopsies [63], plasma [2, 64] and CSF [65]. TNF-α/TNFR-1 complexes can enhance the cytokine’s action in chronic inflammatory conditions by its slow release [66].

CSF TNFR-1 was significantly higher in ALS patients than controls, independent of age and sex, consistent with prior reports of proportional increases in TNF-α/TNFR pathway activity with disease duration in plasma and CSF [2, 64, 65] and with a proposed “switch” in TNF receptor localization from neurons to microglia over the disease course [67]. In a twin study, peripheral blood cells from the ALS-affected twin spontaneously secreted TNF-α, a phenomenon not observed in the healthy twin [68]. Our data do not support an independent association between TNFR-1 and disease severity or progression rate. The age-related rise in CSF TNFR-1 we observed parallels reports of age-related increases in serum TNFR-1 in other conditions [69] and is consistent with an age-related shift of microglia toward a pro-inflammatory phenotype [11].

The association between TNFR-1 and bulbar onset should be treated as exploratory given the small subgroup size. Differences in cytokine levels among ALS subtypes correlate with survival, suggesting specific immune responses [70], but we found no studies regarding specifically TNFR-1 and the type of ALS.

Given the assay limitations for TNF-α, future studies should measure TNF-α, TNFR-1 and TNFR-2 together with a more sensitive method to clarify their relative contributions.

## Conclusions

CSF TNFR-1 concentrations were higher in ALS patients than in controls, independent of age and sex, and TNFR-1 discriminated the two groups with fair accuracy in an exploratory, non-validated analysis. We interpret this as evidence that CSF TNFR-1 is associated with a diagnosis of sporadic ALS and is a candidate disease-associated inflammatory biomarker, rather than as evidence of validated diagnostic performance, causality, or a specific pathophysiological mechanism linking peripheral and central inflammation. The strong plasma–CSF CRP correlation we observed was not ALS-specific.

The relationship between peripheral inflammation markers (represented by peripheral blood inflammatory markers) and CNS inflammation markers (represented by proinflammatory cytokines and CRP in CSF) is ambiguous and requires further, more detailed studies. Given that the immune response alters with age and that the average onset of ALS is in the sixth decade of life, it is plausible that immune system dysfunction contributes to pathogenesis. Longitudinal studies with appropriate disease controls, independent validation of any candidate cut-off, and direct measures of blood–CSF barrier integrity are needed before CSF TNFR-1 or related markers can be used for diagnostic, prognostic, or mechanistic purposes.

## Limitations

The study and control groups differed significantly in age, a potential confounder given the age-related rise in TNFR-1; this was addressed with a multivariable model (Sect.  3.3), which did not alter the primary conclusion, but residual confounding cannot be excluded. The study group consisting of 45 patients, while modest, is consistent with sample sizes typically reported for this disease, due to its rarity and short survival. The ALS cohort was drawn from a clinical trial population restricted to patients aged ≤ 65 years, which limits generalizability to the broader ALS population, whose peak incidence occurs between ages 50 and 70. Owing to the invasive nature of the lumbar puncture, the control group comprised ER patients instead of disease controls undergoing differential diagnosis for motor neuron disease.

Methodological limitations may also be significant. Due to the limitations of the immunofluorescence method used, TNF-α levels were below the lower limit of quantification, precluding any biological conclusion about this cytokine; future studies should use a more sensitive immunoenzymatic method. Lastly, the bulbar-onset association with lower TNFR-1 is based on a small subgroup (*n* = 10) and reached significance only in an adjusted model, not in the univariate comparison, and should be treated as hypothesis-generating rather than confirmatory.

## Data Availability

Source data are available upon request, by contacting the corresponding author. Role of artificial intelligence in creating content. Artificial intelligence has not been used to create this article.
